# The overlaying oil type influences *in vitro* embryo production: differences in composition and compound transfer into incubation medium between oils

**DOI:** 10.1038/s41598-017-10989-5

**Published:** 2017-09-05

**Authors:** Cristina A. Martinez, Alicia Nohalez, Inmaculada Parrilla, Miguel Motas, Jordi Roca, Inmaculada Romero, Diego L. García-González, Cristina Cuello, Heriberto Rodriguez-Martinez, Emilio A. Martinez, Maria A. Gil

**Affiliations:** 1 Faculty of Veterinary Medicine, International Excellence Campus for Higher Education and Research “Campus Mare Nostrum”, University of Murcia, Murcia, Spain; 2 Institute for Biomedical Research of Murcia (IMIB-Arrixaca), Murcia, Spain; 3 Instituto de la Grasa, (CSIC), Campus University Pablo de Olavide, Sevilla, Spain; 4 Department of Clinical & Experimental Medicine (IKE), Linköping University, Linköping, Sweden

## Abstract

The oil overlay micro-drop system is widely used for cultures of mammalian gametes and embryos. We evaluated hereby the effects of two unaltered commercial oils— Sigma mineral oil (S-MO) and Nidoil paraffin oil (N-PO)—on *in vitro* embryo production (IVP) outcomes using a pig model. The results showed that while either oil apparently did not affect oocyte maturation and fertilization rates, S-MO negatively affected embryo cleavage rates, blastocyst formation rates, and, consequently, total blastocyst efficiency of the system. No differences in the oxidation state were found between the oils or culture media incubated under S-MO or N-PO. Although both oils slightly differed in elemental composition, there were no differences in the concentrations of elements between fresh media and media incubated under oils. By contrast, we demonstrated clear oil-type differences in both the composition of volatile organic compounds (VOC) and the transfer of some of these VOC´s (straight-chain alkanes and pentanal and 1,3-diethyl benzene) to the culture medium, which could have influenced embryonic development.

## Introduction

Mammalian oocytes and embryos are usually cultured in microdrops under an oil overlay, which helps to maintain the stability of pH and osmolality in the culture medium^1–4^, thus minimizing adverse metabolic effects on the embryo/s^5, 6^ and the eventual DNA and protein alterations that osmotic stress causes^7^. Further beneficial effects of oil overlay include the removal of accumulated lipophilic toxic substances from the medium^3, 8^, similarly to how some steroids (progesterone and estradiol) are absorbed by the oil during oocyte maturation^9–12^.

By contrast, the use of an oil overlay may also exert adverse effects by acting as a source of contamination in the incubation media^8, 13^ and, therefore, for the gametes and embryos. In this context, two situations should be defined. The first is the use of oils containing undetected contamination, which has long been recognized as a problem for embryo cultures. Several reports have shown compromised embryo development caused by batches of oils contaminated with peroxides^14–16^, alkenals and aldehydes^16^, Triton X-100^16^ and zinc^17^. The second is the use of different unaltered oils, which can exert different effects on embryonic development. Despite the large number of commercially available oils, few studies have directly compared their effectiveness in terms of *in vitro* embryo production (IVP) outcomes. Mineral oil (MO) is obtained by the fractional distillation of crude oil and contains complex mixtures of straight-chain hydrocarbons and some aromatic hydrocarbons and unsaturated hydrocarbons^13^. The oil typically used for porcine IVP cultures is mineral oil from Sigma-Aldrich Co (S-MO). Although MO is also commonly known as paraffin oil (PO), some enterprises claim that PO contains more saturated hydrocarbons than MO and is therefore more resistant to peroxidation. That is the case of Nidoil^TM^ (Nidacon) (N-PO), a light paraffin oil widely used as an overlay during gamete and embryo culture or manipulation in rodents and humans. The available information, although scarce and limited to only a few species, indicates that unaltered MO and PO influence culture systems differently. In mouse^18^, bovine^4^ and human^19^ IVP systems, the use of PO enhances embryonic development compared with MO. Although these results suggest that differences in the chemical composition of the oils might be related to the final IVP efficiency, information on the compositions of these oils, including inorganic elements and volatile organic compounds (VOCs), and their impact on embryonic development is lacking. Furthermore, the possible transfer of some of these compounds from the oil into the culture medium has also not been reported. The aims of this study were 1) to compare the effectiveness of unaltered S-MO and N-PO on IVP outcomes using the pig as a model, 2) to evaluate the oxidation levels of the oils and media throughout the culture periods, 3) to determine differences in the elemental composition and VOCs between these types of oils, and 4) to investigate the transfer of compounds into the culture medium from the respective oils.

## Results

### IVP outcomes

**Figure 1:**
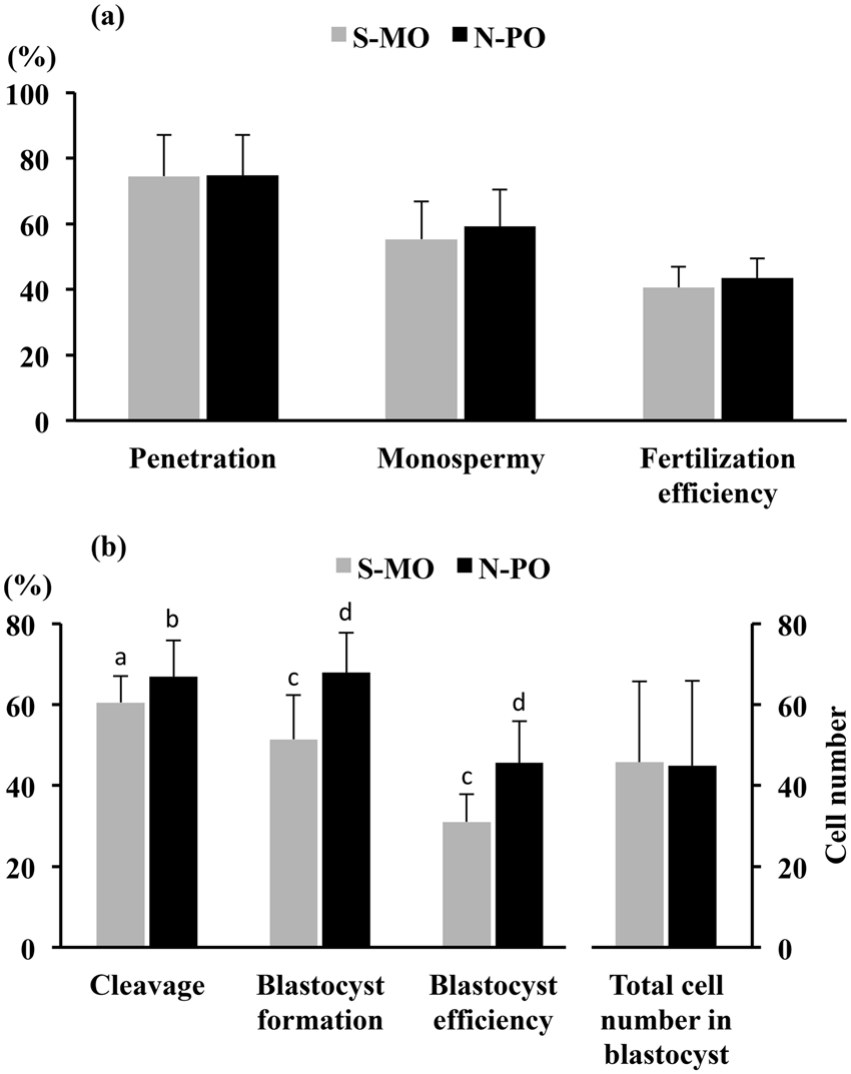
Porcine IVP using two types of oil overlay. (**a**) Fertilization parameters: Oocytes were matured and fertilized in culture media overlaid with Sigma mineral oil (S-MO; n = 189) or Nidoil paraffin oil (N-PO; n = 176). There were no differences in the penetration rate (percentage of penetrated oocytes/total inseminated oocytes), monospermy (percentage of oocytes containing only one male pronucleus/total of oocytes penetrated) and fertilization efficiency (percentage of monospermic oocytes/total of oocytes inseminated) between oils. The data are presented as the mean ± SD of four replicates. (**b**) Embryonic development: After gamete co-incubation, the presumed zygotes were cultured in glucose-free embryo culture medium supplemented with pyruvate and lactate for 2 days and then in fresh embryo culture medium containing glucose for 5 additional days. The cultures were maintained under S-MO (n = 302) or N-PO (n = 305). The different letters within each variable indicate significant differences (a, b: P < 0.05; c, d: P < 0.004). The data are presented as the mean ± SD of four replicates.

This study was conceived of because in our routine experiments we noticed that the use of N-PO overlay performed always better than S-MO overlay in terms of cleavage rates, blastocyst formation and blastocyst production efficiency, regardless of the lot of oil used (Supplementary tables S1 and S2). In this experiment, we compared the effects of both types of oil overlays (S-MO: Cat. no. M8410, lot no. MKBX6122V, Sigma-Aldrich and N-PO: Cat. no. NO-100, lot no. 300NOWF21-1, Nidoil^TM^, Nidacon) on *in vitro* maturation (IVM), *in vitro* fertilization (IVF) and further development to blastocyst. Both oils had performed well in previous independent experiments. To evaluate the percentage of oocytes that achieved the metaphase-II (MII) stage, immature oocytes were randomly incubated for 44 h in IVM medium covered with S-MO (n = 282) or N-PO (n = 251). In addition, 972 immature oocytes were matured, fertilized and cultured under S-MO or N-PO overlay. Some presumptive zygotes (n = 365) were fixed and stained at 18 h post-insemination to determine the fertilization parameters (i.e., penetration, monospermy and fertilization efficiency). The remaining presumed zygotes were incubated in culture medium covered with S-MO (n = 302) or N-PO (n = 305) for seven days to assess the efficiency of embryonic development. All blastocysts formed at day 7 were stained for cell counting. The results showed that high proportions of the oocytes achieved the MII stage at 44 h of maturation, with no differences between oil groups (82.1 ± 5.7% and 82.4 ± 4.8% for the S-MO and N-PO groups, respectively). Similarly, the type of oil overlay did not affect the fertilization parameters. The penetration and monospermic rates were approximately 75% and 55%, respectively, and the total efficiency of fertilization was close to 40% in both groups (Fig. 1a). However, presumed zygotes developed differently into the 2- to 4-cell stage, depending on the type of oil used. The proportion of cleaved embryos was higher (P < 0.04) in the N-PO group (66.9 ± 9.0%) than in the S-MO group (60.5 ± 6.5%) at 48 h post-insemination. Moreover, a higher proportion (P < 0.004) of cleaved embryos cultured under N-PO achieved the blastocyst stage (67.9 ± 9.9%) compared to those incubated under S-MO (51.4 ± 10.9%). These results were consistent across the four replicates. Consequently, the total blastocyst efficiency (i.e., the percentage of blastocysts formed from the total number of oocytes cultured) was almost 15 points higher in the N-PO group than in the S-MO group (45.6 ± 10.3% and 31.0 ± 6.8%, respectively) (P < 0.001) (Fig. 1b). Nevertheless, the blastocysts produced under both types of oil were morphologically indistinguishable, and they had similar numbers of cells (45.7 ± 20.0 and 44.9 ± 20.9 cells per blastocyst for the S-MO and N-PO groups, respectively).

### Oxidation levels

**Figure 2:**
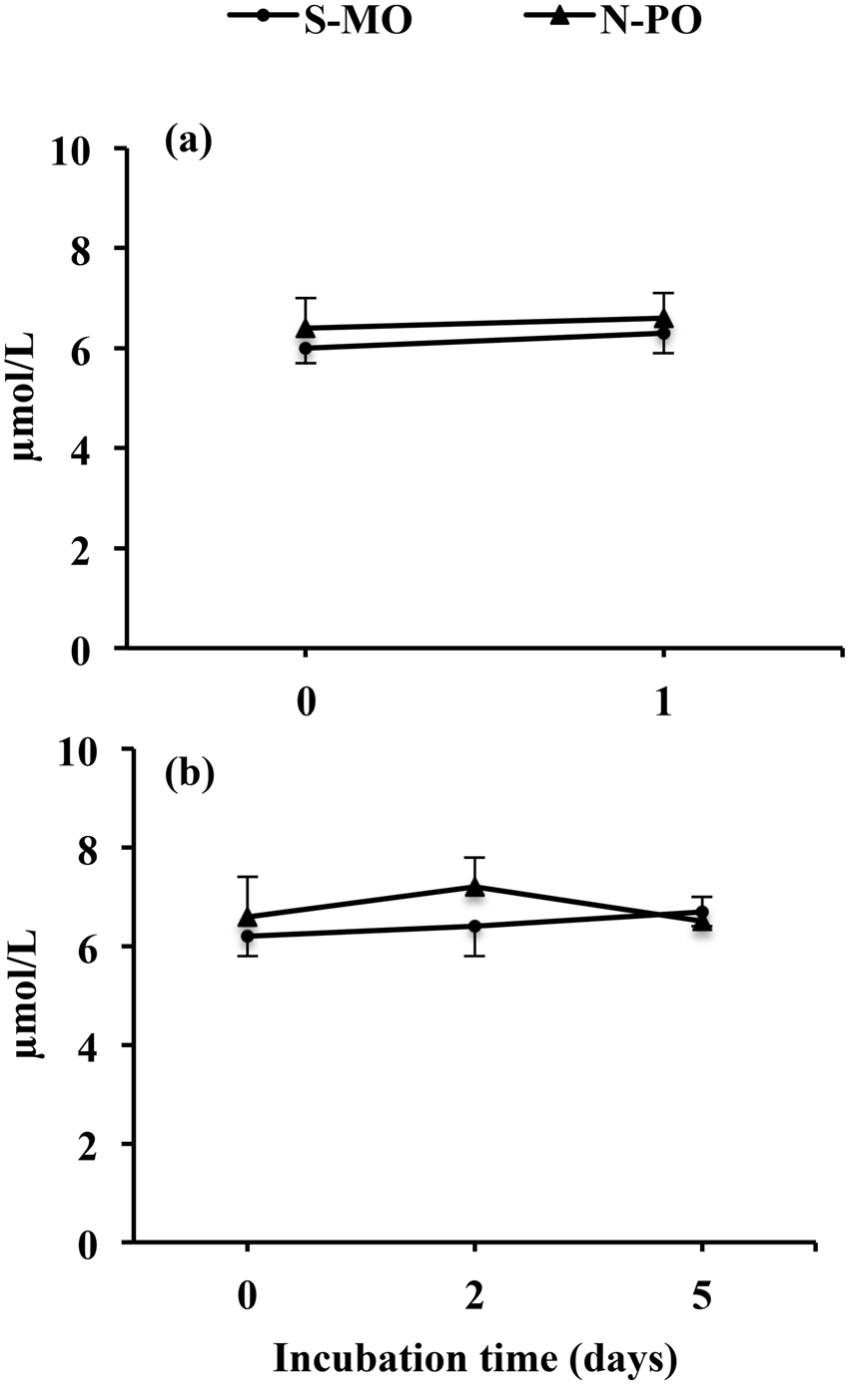
POVs in oils incubated with IVM and embryo culture media. Drops of IVM or embryo culture medium were covered with Sigma mineral oil (S-MO) or Nidoil paraffin oil (N-PO) and incubated for 0 and 1 day (IVM medium) (**a**) or for 0, 2 and 5 days (embryo culture medium) (**b**) under the same conditions used for IVM or embryo culture but without oocytes or embryos. At the end of the incubation, the oil overlay was removed with a micropipette and transferred to 5-mL tubes for the analysis. The POVs of both oils were below the recommended maximum value (8.5 µmol/L), and these levels did not vary throughout the incubations. The data are presented as the mean ± SD (three replicates).

**Figure 3:**
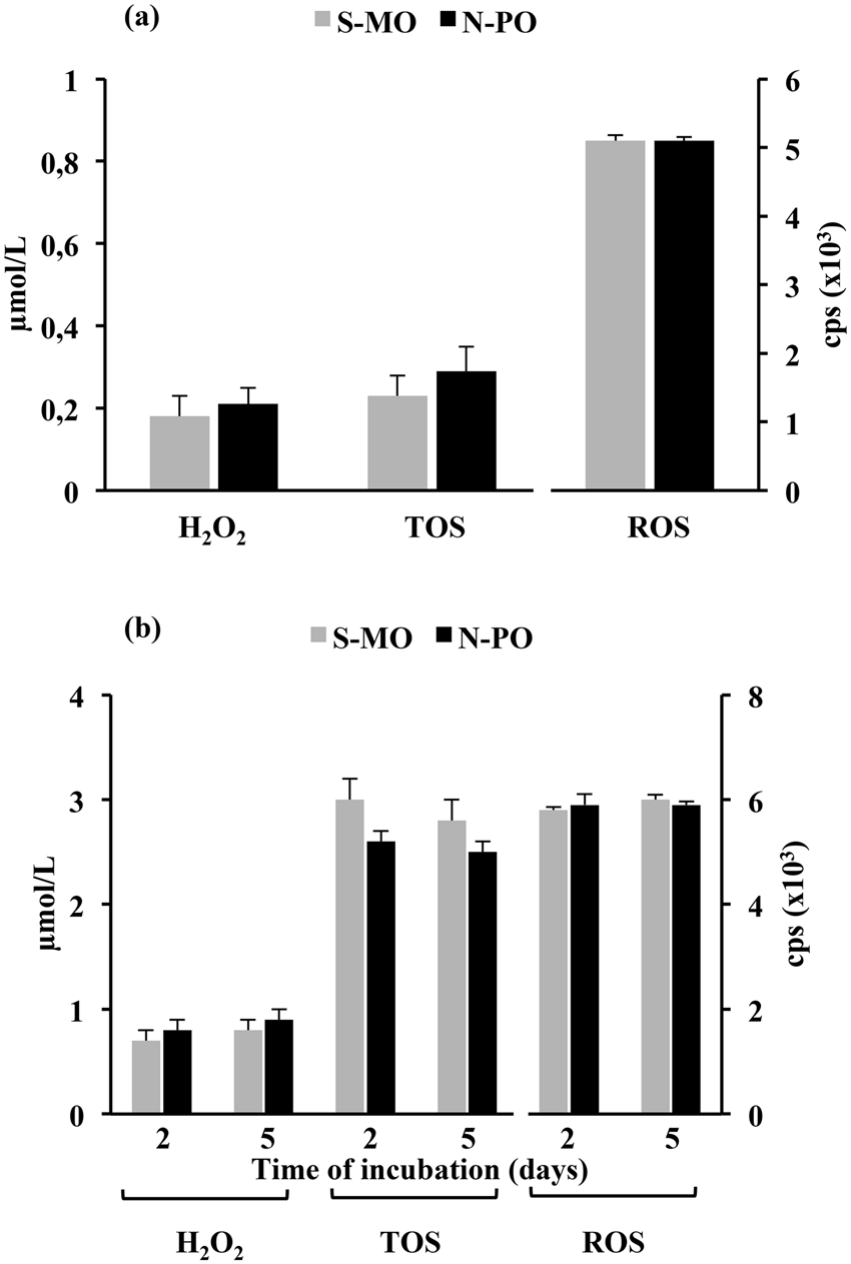
Oxidation values of the culture media incubated under mineral oil and paraffin oil. Drops of IVM or embryo culture medium were covered with Sigma mineral oil (S-MO) or Nidoil paraffin oil (N-PO) and incubated for 1 day (IVM medium) (**a**) or for 2 and 5 days (embryo culture medium) (**b**) under the same conditions used for IVM or embryo culture but without oocytes or embryos. At the end of the incubation, the media were carefully removed and placed in a new Petri dish. This process was repeated three times using a new pipette every time to avoid cross-contamination with the oil. The analysis was performed immediately after sample collection. The media incubated under S-MO and N-PO had similar levels of hydroperoxides, ROS and TOS, regardless of the medium and incubation time used. The data are presented as the mean ± SD (three replicates).

To determine the oxidation status of both types of oils and media, drops of IVM or embryo culture media were covered with S-MO or N-PO and incubated for 1 day (IVM medium) or 2 and 5 days (embryo culture medium) under the same conditions used for IVM or embryo culture but without oocytes or embryos. At the end of the incubation, the oil overlay was removed with a micropipette and transferred to 5-mL tubes for analysis. The medium was carefully removed and placed in a new Petri dish. This process was repeated three times using a new pipette each time to avoid cross-contamination with the oil. Non-incubated oils were also collected for analysis. The analysis was performed immediately after sample collection. The peroxide values (POVs) in both non-incubated oils were below the recommended maximum level (8.5 µmol/L), and these levels did not vary throughout the incubations (Fig. 2). The media incubated under S-MO or N-PO had similar levels of hydrogen peroxide (H_2_O_2_), reactive oxygen species (ROS) and total oxidation status (TOS), regardless of the media and incubation times used (Fig. 3).

### Inorganic elements

The elemental compositions of S-MO, N-PO and embryo culture medium under oil overlays were measured in incubated (5 days) oils and media in the absence of oocytes. Fresh embryo culture medium was also measured. The collected oil and medium samples were transferred to 5-mL polystyrene round-bottom tubes (BD Falcon, BD Biosciences, Erembodegem, Belgium) and subjected to inductively coupled plasma optical emission spectrometry (ICP-OES) analysis on the day of sample collection. The assay was controlled by applying the method to the Certified Reference Material (CRM) EnviroMAT HU-1 Used oil (SCP Science, Canada, obtained from QMX Laboratories, Essex, UK). The results obtained from analyzing the CRM with ICP-OES and the certified values are shown in Table 1. The elemental concentrations determined by ICP-OES fell within the certified tolerance interval. Since this method achieved good precision in the CRM analysis, ICP-OES was applied for the elemental analysis of both types of oils and the culture medium. The results are shown in Table 2. The concentrations of As, Be, Bi, Cd, Co, Li, Mo, Sb, Se and Tl were under the limits of quantification of ICP-OES. Additionally, Cd and Se were not detected using ICP mass spectrometry (data not shown). The analytical results indicated that the elemental compositions of S-MO and N-PO varied slightly, with N-PO containing more Na, Ti, Al and S than S-MO (P < 0.03). The values for the remaining elements analyzed were similar for both oils. S-MO and N-PO contained higher concentrations of Cu and Pb than fresh culture medium (P < 0.05). However, the concentrations of these elements in fresh culture medium did not vary relative to those found in incubated culture medium, regardless of the type of oil used for overlay. There were also no differences in the concentrations of the remaining elements between fresh culture medium and culture medium incubated under S-MO and N-PO.

**Table 1:** Inorganic element concentrations in the certified reference material (EnviroMAT HU-1 Used oil).

Element	Present analysis (mg/kg)*	Reference values (mg/kg)		
		Consensus value	Confidence interval 95%	Tolerance interval
Ca	64.2 ± 21.4	72	67–77	43–101
Mg	12.7 ± 1.1	11	10–12	6–16
Cr	13.8 ± 0.3	15	13–17	6–24
Cu	2648.8 ± 875.3	3132	2906–3358	1854–4410
Fe	53.6 ± 1.1	59	53–65	26–92
Mn	17.8 ± 0.4	18	17–19	13–23
Ni	55.0 ± 1.2	45	42–48	29–61
Pb	22.3 ± 2.2	20	19–21	13–27
Ti	7.0 ± 0.4	9	7–11	0–20
Zn	17.9 ± 1.7	16	14–18	3–29
Al	20.8 ± 1.6	14	11–17	0–31

*Values are means ± SD (four replicates).

**Table 2:** Inorganic element concentrations in 5 days incubated mineral oil, paraffin oil, and embryo culture medium covered with mineral and paraffin oils.

Element (mg/kg)	Sigma mineral oil	Nidoil paraffin oil	Fresh medium (non incubated)	Medium incubated under Sigma mineral oil	Medium incubated under Nidoil paraffin oil
Ca	4.0 ± 1.2	4.2 ± 0.7	63.1 ± 2.7^#^	65.9 ± 1.7	65.6 ± 2.8
Na	30.0 ± 14.4*	53.0 ± 7.2	2433.6 ± 124.8^#^	2552.1 ± 148.8	2480.3 ± 104.5
K	0.7 ± 0.4	1.2 ± 0.6	242.4 ± 11.7^#^	233.4 ± 14.8	223.1 ± 10.1
Mg	0.4 ± 0.1	0.3 ± 0.1	27.1 ± 1.5^#^	26.2 ± 1.8	24.7 ± 1.1
Rb	0.04 ± 0.01	0.05 ± 0.01	0.04 ± 0.01	0.04 ± 0.01	0.05 ± 0.01
Sr	0.02 ± 0.01	0.03 ± 0.04	0.01 ± 0.01	0.01 ± 0.01	0.01 ± 0.01
Cr	0.07 ± 0.05	0.03 ± 0.01	0.07 ± 0.04	0.05 ± 0.04	0.10 ± 0.40
Cu	0.2 ± 0.1	0.5 ± 0.2	0.04 ± 0.03^#^	0.05 ± 0.04	0.1 ± 0.2
Fe	1.4 ± 1.6	0.9 ± 0.3	0.9 ± 0.5	0.6 ± 0.1	1.1 ± 0.4
Mn	0.03 ± 0.02	0.01 ± 0.01	0.2 ± 0.01^#^	0.2 ± 0.01	0.2 ± 0.04
Ni	0.1 ± 0.05	0.1 ± 0.04	0.04 ± 0.04	0.02 ± 0.01	0.04 ± 0.4
Pb	0.05 ± 0.02	0.05 ± 0.01	0.02 ± 0.01^#^	0.02 ± 0.02	0.02 ± 0.1
Ti	0.1 ± 0.1*	0.6 ± 0.2	1.4 ± 0.7^#^	1.2 ± 0.9	1.5 ± 0.5
Zn	0.3 ± 0.1	0.1 ± 0.04	0.1 ± 0.05	0.1 ± 0.1	0.3 ± 0.2
Al	1.0 ± 0.4*	1.9 ± 0.4	1.5 ± 0.5	1.2 ± 0.6	1.3 ± 1.1
S	2.4 ± 0.9*	6.9 ± 0.7	465.5 ± 14.8^#^	452.4 ± 12.8	443.0 ± 17.8

*Indicate differences (P < 0.03) between Sigma mineral and Nidoil paraffin oils. # Indicate differences (P < 0.05) between fresh culture medium and Sigma mineral and Nidoil paraffin oils. There were no differences in element concentrations between fresh culture medium and incubated culture medium covered with Sigma mineral or Nidoil paraffin oil. Values are means ± SD (four replicates).

### VOCs

The differences in the VOC compositions of the S-MO, N-PO and embryo culture medium were characterized, and whether or not these compounds could be transferred to the medium was investigated. For this purpose, we utilized a design similar to that described for the elemental evaluation. At the end of the incubation, the oils and culture medium were collected and transferred into headspace screw-top vials (Agilent Technologies, Böblingen, Germany). The vials were tightly crimped and stored at 4 °C until VOC analysis, which was performed 24 h later. Solid-phase microextraction (SPME) sampling was performed, followed by gas chromatography (GC) separation with flame ionization detection (FID), which produced chromatograms containing more than 100 appreciable peaks. Table 3 presents the concentration of VOCs determined in S-MO and N-PO and the medium before and after being in contact with the oils. The major VOCs that were unambiguously identified in the oils were alkanes, with the exception of pentanal and 1,3-diethyl benzene, which were identified exclusively in the S-MO. The transfer rates of VOCs from the oils to the culture medium depended on the type of compound. Thus, while most of the straight-chain alkanes were clearly transferred to the culture medium, with transfer rates ranging from 30% to 50%, the branched-chain alkanes were not transferred, regardless of the type of oil analyzed. The only non-alkanes identified in our study (i.e., pentanal and 1,3-diethyl benzene) were clearly transferred (approximately 100%) to the culture medium.

**Table 3:** Volatile compounds determined in 5 days incubated mineral oil, paraffin oil, and embryo culture medium covered with mineral and paraffin oils.

Compounds (mg/kg)	Sigma mineral oil	Nidoil paraffin oil	Fresh medium (non incubated)	Medium incubated under Sigma mineral oil	Medium incubated under Nidoil paraffin oil
Heptane	0.4 ± 0.01	6.2 ± 0.7	trace	trace	trace
Pentanal	0.3 ± 0.7	n.d.	n.d.	0.2 ± 0.1	n.d.
4-ethyl heptane	0.1 ± 0.01	n.d.	n.d.	n.d.	n.d.
4-methyl decane	0.05 ± 0.01	0.5 ± 0.02	n.d.	n.d.	n.d.
2-methyl decane	0.2 ± 0.02	n.d.	n.d.	n.d.	n.d.
3-methyl decane	0.1 ± 0.01	n.d.	n.d.	n.d.	n.d.
1,3-diethyl benzene	0.1 ± 0.1	n.d.	n.d.	0.1 ± 0.1	n.d.
4-methyl heptane	n.d.	3.9 ± 0.9	n.d.	n.d.	n.d.
Octane	n.d.	6.0 ± 0.2	n.d.	n.d.	1.8 ± 0.01
2-methyl octane	n.d.	3.2 ± 0.3	n.d.	n.d.	n.d.
3-methyl nonane	n.d.	0.4 ± 0.01	n.d.	n.d.	n.d.
Decane	n.d.	2.3 ± 0.1	n.d.	n.d.	0.7 ± 0.01
Dodecane	n.d.	1.4 ± 0.1	n.d.	n.d.	0.5 ± 0.01
Tetradecane	n.d.	3.3 ± 0.1	trace	n.d.	1.2 ± 0.03
Pentadecane	n.d.	0.6 ± 0.1	n.d.	n.d.	0.3 ± 0.1
Hexadecane	trace	4.2 ± 0.1	n.d.	n.d.	1.4 ± 0.05
Heptadecane	n.d.	0.9 ± 0.1	n.d.	n.d.	0.4 ± 0.03
Octadecane	n.d.	0.4 ± 0.3	trace	trace	0.2 ± 0.03

n.d., not detected. The values are expressed as mean ± SD (four replicates).

## Discussion

Three main findings are reported in the present study. As in other species, the type of oil cover used during IVM, IVF and embryo culture affected porcine embryo production. Although S-MO and N-PO differ in their elemental composition, only two elements (Cu and Pb) were more abundant in the oils than in the culture medium. However, the values of these and other elements analyzed in this study were similar in both the fresh culture medium and that incubated under S-MO or N-PO, suggesting that no elemental interactions occurred between the oil overlays and the medium. We also demonstrated the transfer of VOCs from the oil overlay to the medium, which could affect embryonic development.

Our results indicated that S-MO and N-PO were equally able to sustain nuclear maturation and fertilization, although embryonic development at days 2 and 7 differed significantly. N-PO performed better than S-MO and resulted in increased embryo cleavage and blastocyst formation rates and, therefore, higher total efficiency of the system. These results confirm those obtained in mouse^18^, bovine^4^ and human^19^ studies and demonstrate, for the first time, that the type of oil used to cover the culture drops significantly influences the *in vitro* development of porcine embryos. Although the available literature is limited, N-PO has been suggested to contain more saturated hydrocarbons than S-MO and, therefore, is considered more resistant to peroxidation. Because oil overlay POVs between 0.5 and 1.0 mEq/kg cause the death of all embryos by day 2 or 3 of culture^14^, we speculated that differences in the peroxide levels during culturing may be responsible for the differences in blastocyst outcomes between oils. However, this speculation is inconsistent. In our study, the peroxidation index values in both oils were lower than 0.02 mEq/kg (<8.5 µmol/L) at 0 h, 48 h and 5 days of culture. Moreover, no differences in the hydroperoxide, ROS and TOS levels of the culture medium incubated for the same periods under S-MO or N-PO were found, indicating that oxidative stress does not play a role in differences in embryonic development when unaltered oils are used.

Although the oils used to overlay culture media can vary in quality, the reasons why different oil types affect embryonic development differently remain unclear. However, two main mechanisms should be considered to explain these differences. First, the oil may interact with lipophilic compounds in the medium and thus modify its composition. Indeed, oil overlays have been demonstrated to alter the amounts of fatty acids in different lipid classes, which could modify the embryo membrane composition and affect the development and freezability of the embryos^20^. Moreover, oil overlays can extract steroid hormones, which are secreted by cumulus cells and oocytes, from the maturation media^11, 12^. Although no data are available, differences in the absorption of lipophilic compounds by different types of oils seem unlikely. Second, embryo-toxic compounds present in the oil may be transferred to the culture medium and affect embryonic development. Because zinc was identified as a toxic compound in a batch of oil^17^, we decided to examine the potential importance of heavy metals and other elements to identify differences between the two types of oil used. Importantly, we found that the culture medium was contaminated with Rb, Sr, Cr, Cu, Fe, Mn, Ni, Pb, Ti, Zn and Al, most of which can induce DNA damage through oxidative stress^21–25^. Although the source of such contamination is unknown, albumin used for medium supplementation could be involved, since protein supplements normally contain significant levels of trace metals^26^. All elements analyzed were present at similar concentrations in both oils, except Na, Ti, Al and S, which were 2- to 7-fold higher in N-PO than in S-MO. However, these elements were present in the oils at concentrations similar to (i.e., Al) or less than (i.e., Na, Ti and S) those found in the fresh culture medium, which might exclude them as causative factors. Cu and Pb were the only elements whose concentrations were higher in the oils than in the medium (10-fold and 2.5-fold higher, respectively). Cu has been shown to be toxic to embryos when it is added to mouse embryo culture medium at very high concentrations (≥50 µM). However, lower Cu-concentrations (1 to 10 µM) did not affect blastocyst formation in mouse^27^ or even increase the blastocyst formation rate by decreasing apoptotic blastomeres in bovine blastocysts^28^. These Cu concentrations were above the levels found in our study in both the medium (~0.05 µM) and the oils (~1.0 µM). Pb has been shown to affect the control of both meiosis arrest and meiosis resumption of mouse oocytes^29^ and to inhibit oocyte growth and embryonic development in large ruminants^30^. However, these effects were observed at concentrations 10-fold higher than those estimated in our study in both the oils and culture medium. In the present study, the values for all elements analyzed were similar in the culture medium before and after incubation under S-MO and N-PO. This finding indicates none of these elements was incorporated into the medium from the oil during five days of incubation. Together with the observations mentioned above, this finding suggests that inorganic elements might be excluded as causative factors underlying the differences in embryonic development between oils.

Live organisms produce many VOCs via their metabolic processes^31^. In our study, the incubation of medium under oil was performed in the absence of oocytes or embryos. Therefore, the VOCs were identified in the absence of metabolic processes. Although the possibility of contamination with exogenous compounds must be considered, we can assume that most of the VOCs identified in the present study were endogenous. Two facts support this assumption. First, our data showed that most of the alkanes were detected in the culture medium incubated under an oil overlay but not in the fresh culture medium that had not come into contact with the oil. Second, most of the alkanes identified in the N-PO were also identified in the medium incubated under N-PO but not in the medium incubated under S-MO. Our results suggest that the compositions of S-MO and N-PO may vary widely. However, our ultimate goal was not to determine the VOC compositions of the oils but, rather, to evaluate the possibility that VOCs may be transferred from oil overlays into the culture medium during incubation. To the best of our knowledge, this is the first report demonstrating that some VOCs are introduced into the incubation medium by the oil overlay. Thus, we cannot compare our findings with available literature data. Interestingly, most of the identified straight-chain hydrocarbons in the oils were transferred to the medium after five days of incubation with transfer rates ranging from 30% to 50%. By contrast, none of the branched hydrocarbons was transferred into the medium, regardless of the type of oil analyzed. In addition, the oils clearly differed in terms of the presence of aldehydes (pentanal) and aromatic hydrocarbons (1,3-diethyl benzene). These compounds were identified exclusively in the S-MO, and in both cases, the transfer rate to the medium was close to 100%. These findings indicate that oil overlays can alter the compositions of culture media by transferring certain types of compounds.

In conclusion, the type of unaltered commercial oil used to overlay maturation, fertilization and embryo culture media can greatly influence the cleavage and blastocyst formation rates and, therefore, the porcine IVP. Our results show that the blastocyst efficiency rates in the cultures under S-MO were decreased by 1.5-fold compared with those in the cultures under N-PO. Under those terms we conclude that N-PO performs better than S-MO for porcine IVP systems. Unfortunately, despite significant research, we can not define which factors/compounds/molecules were implicated. Although the elemental compositions of S-MO and N-PO varied, the results of the present study do not indicate presence of elemental interactions between the oil overlay and the culture medium, suggesting that the ionic elements analyzed in our study were not responsible for the differences observed in embryonic development between oils. On the other hand, we demonstrated clear differences in the composition of VOCs between oils and the transfer of VOCs (straight-chain alkanes and pentanal and 1,3-diethyl benzene) to the culture medium, which could have influenced blastocyst development.

## Methods

All experimental procedures used in this study were performed in accordance with the 2010/63/EU EEC Directive for animal experiments and were reviewed and approved by the Ethical Committee for Experimentation with Animals of the University of Murcia, Spain (research code: 183/2015).

### Reagents and culture media

All chemicals used in this study were purchased from Sigma-Aldrich Co. (Alcobendas, Madrid, Spain) unless otherwise indicated.

The medium used for the collection of oocytes-cumulus complexes (COCs) was a Tyrode’s lactate-4-(2-hydroxyethyl)−1-piperazineethanesulfonic acid (HEPES)-polyvinyl alcohol (PVA) medium^32^ with some modifications^33^. This medium contains 2-mM NaHCO_3_, 10-mM HEPES and 0.1% (w/v) PVA. The sperm-washing medium was Dulbecco’s phosphate-buffered saline supplemented with 4-mg/mL bovine serum albumin (BSA), 0.34-mM sodium pyruvate and 5.4-mM D-glucose (mDPBS)^34^. The IVM medium was TCM-199 (Gibco Life Technologies S.A. Barcelona, Spain) supplemented with 0.57-mM cysteine, 0.1% (w/v) PVA and 10-ng/mL epidermal growth factor. The basic medium used for IVF was a modified Tris-buffered medium^35^ supplemented with 2.0-mM caffeine and 0.2% BSA. The embryo culture medium was North Carolina State University^36^ with 0.4% BSA.

### Oocyte collection and IVM

Ovaries from prepuberal gilts were collected from a local slaughterhouse and transported to the laboratory at 35 °C within 1 h after collection in 0.9% NaCl containing 70-g/mL kanamycin. The COCs were collected with a surgical blade from the surfaces of intact healthy antral follicles with diameters of 3 to 6 mm. Oocytes with a compact cumulus mass and dark, evenly granulated cytoplasm were selected and washed three times in IVM medium. Groups of 40 to 50 COCs were transferred into each well of a 4-well multidish (Nunc, Roskilde, Denmark) containing 500 µL of pre-equilibrated IVM medium supplemented with 10-IU/mL equine chorionic gonadotrophin (Folligon, Intervet International B.V., Boxxmeer, The Netherlands) and 10-IU/mL human chorionic gonadotrophin (Veterin Corion, Divasa Farmavic, S.A., Barcelona, Spain), and cultured for 22 h. Next, the COCs were incubated for another 22 h in IVM medium without hormones. Maturation was performed at 38.5 °C in 5% CO_2_ in air and 97% relative humidity.

### IVF

After IVM, the cumulus cells were removed with 0.1% hyaluronidase in IVM medium by vortexing for 2 min at 1660 rounds/min. The denuded oocytes were washed three times in IVM medium and another three times in IVF medium and were then inseminated as described by Gil *et al*.^37^. Briefly, groups of 40 to 50 oocytes were placed in 50-µL drops of pre-equilibrated IVF medium in a 35 mm × 10 mm Petri dish (Falcon, Becton Dickinson Labware, Franklin Lakes, USA) and held at 39 °C in an atmosphere of 5% CO_2_ in humidified air for 30 min until the addition of spermatozoa.

Semen from a mature Pietrain boar was processed and cryopreserved in 0.5-mL straws as described by Roca *et al*.^38^. For each replicate, one pool of semen was made from two straws thawed in a circulating water bath at 37 °C for 20 s. Next, 100 µL of thawed semen was washed three times by centrifugation at 1900 × g for 3 min in mDPBS. The resulting pellet was re-suspended in IVF medium, and after the appropriate dilution, 50 µL of the sperm suspension was added to the 50-µL drop of IVF medium containing the oocytes. The spermatozoa:oocyte ratio was 1000:1 (300,000 spermatozoa/mL). The gametes were co-incubated at 39 °C in a humidified atmosphere of 5% CO_2_ in air for 5 h.

### *In vitro* embryo culture

After gamete co-incubation, the presumed zygotes were washed by mechanical pipetting three times in pre-equilibrated embryo culture medium to remove spermatozoa not bound to the zona pellucida and transferred (30 zygotes/well) to a 4-well multidish, with each well containing 500 µL of glucose-free embryo culture medium supplemented with 0.3-mM pyruvate and 4.5-mM lactate for 2 days and then in fresh embryo culture medium containing 5.5-mM glucose for 5 additional days. Cultures were maintained at 39 °C, 5% CO_2_ in air, and 97% relative humidity.

### Assessment of the maturation, fertilization and embryonic development parameters

To evaluate the IVM and IVF parameters, the oocytes and presumed zygotes were mounted on slides, fixed for 48 to 72 h in 25% (v:v) acetic acid in ethanol at room temperature, stained with 1% lacmoid in 45% (v:v) acetic acid, and examined under a phase-contrast microscope at 400 × magnification. The IVM rate was assessed at 44 h of maturation. The oocytes were considered mature when their chromosomes were at MII, and they had an extruded first polar body. The IVF parameters were evaluated 18 h after insemination. Oocytes were considered penetrated when they contained one or more swollen sperm heads and/or male pronuclei with their corresponding sperm tails and two polar bodies. The IVF parameters evaluated were as follows: penetration (percentage of penetrated oocytes/total mature oocytes inseminated), monospermy (percentage of monospermic oocytes/total mature oocytes penetrated), and efficiency of fertilization (percentage of monospermic oocytes/total mature oocytes inseminated). *In vitro* embryonic development was evaluated under a stereomicroscope at 2 and 7 days post-insemination. An embryo that had cleaved to the 2- to 4-cell stage was counted as cleaved, and an embryo with a clear blastocele and good or excellent morphology was defined as a blastocyst. The cleavage rate was defined as the percentage of 2- to 4-cell embryos formed from the total number of oocytes inseminated. The blastocyst formation rate was the percentage of 2- to 4-cell embryos that developed to blastocysts. The blastocyst efficiency rate was the percentage of blastocysts formed from the total number of oocytes cultured. The total cell number, as an indicator of embryo quality, was evaluated by mounting each blastocyst on a slide in 4 µL of diluted Vectashield (Vector Laboratories, Burlingame, CA, USA) containing 10-µg/mL Hoeschst 33342 under a coverslip, followed by fluorescence microscopy examination. The nuclei in each blastocyst were stained with Hoeschst and displayed blue fluorescence, and the total number was counted.

### Oil and incubation media analysis

*Peroxide assay in oil*. The POV was measured following the Official Methods of Analysis of the Association of Official Agricultural Chemists^39^. The oil samples (5 g) were treated with 30 mL of acetic acid-chloroform solution (3:2 v/v) and 0.5 mL of saturated potassium iodide solution and were transferred into the burette of a POV meter (DL 25 Titrator, Mettler-Toledo AG, Greifensee, Switzerland). The titration was allowed to run against a standard solution of sodium thiosulfate (25 g/L). The POV was calculated as milliequivalent peroxide per kg of oil according to the following formula: POV (mEq / kg) = S × N × 1000 / W, where “S” is the volume (mL) of sodium thiosulfate used for titration, “N” is the normality of the sodium thiosulfate solution (N = 0.01), and “W” is the sample weight (g). The POV values were finally expressed in µmol/L.

#### Hydroperoxide, ROS and total oxidant status assays in the media

The detection of hydroperoxide (H_2_O_2_) was based on the horseradish peroxidase (HRP) assay with 3,5,3′5′-tetramethylbenzidine (TBM) as described by Rhee *et al*.^40^. The samples were incubated for 15 min at room temperature in a reaction mixture containing 25 mU of HRP and 0.7-mM TMB in 0.1-M sodium phosphate buffer (pH 6.0). The reaction was terminated by the addition of sulfuric acid (0.5 M), and the original H_2_O_2_ concentration of the sample was calculated based on the molar extinction coefficient of diamine (59000 l/mol/cm) by measuring the absorbance at 450 nm using a microplate reader (Powerwave XS, Biotek instruments, Carson City, NV). The ROS were estimated by a 5-amino-2,3-dihydro-1,4-phthalazinedione (luminol)-mediated chemiluminescence assay^41^. For this purpose, luminol (50 µM) prepared in sodium hydroxide containing HRP (1.2 U/mL) was added to the medium and saline solution. The ROS values were determined using a microplate reader (Victor 2 1420 Multilabel Counter; PerkinElmer, Finland) and expressed in counted photons per second (cps). Each sample was scanned for 20 min. A negative or background control was prepared by adding saline solution instead of sample. The TOS in the medium was measured as described by Erel^42^ using an Olympus AU600 Automatic Chemistry Analyzer. This assay is based on the oxidation of ferrous to ferric ions by lipid hydroperoxides under acidic medium. Thus, the amount of Fe^3+^ formed is proportional to the total peroxide content in the sample. For this assay, 150-μM xylenol orange, 140-mM NaCl and 1.35-M glycerol in 25-mM H_2_SO_4_ solution (pH 1.75) were incubated with the sample for 3 min. Subsequently, 5-mM ferrous ion and 10-mM o-dianisidine in 25-mM H_2_SO_4_ were added to the reaction. The change in absorbance was monitored at 600 nm, and the results were calculated from the standard curve of H_2_O_2_ solutions.

#### Inorganic element analysis

The samples were sent to the Ionomics Service of the Centro de Edafologia y Biologia Aplicada del Segura (Center for Applied Soil Science and Biology of the Segura [CEBAS])-Consejo Superior de Investigaciones Científicas (Spanish National Research Council [CSIC]) (Murcia, Spain), and the content of mineral and elemental ions was analyzed using ICP-OES (ICAP 6500 Duo, Thermo Scientific, with One Fast System). Briefly, all samples were provided to the laboratory in polypropylene tubes and were kept refrigerated until ready for initial sample preparation. Aliquots of oil (1 g) and culture medium (2 g) were accurately weighed and treated with 4 mL of trace mineral-grade nitric acid (69% Suprapur, Merck, Darmstadt, Germany) in Teflon reaction tubes. The reactors were closed and placed inside the microwave digestion system (UltraClave-Microwave Milestone®) and subjected to a 20-min digestion step at a temperature of 220 °C and a pressure of 100 bar. Digested samples were then diluted to a final volume of 10 mL with ultrapure water. The limits of quantification were calculated as ten times the standard deviation of 10 blank measurements divided by the slope of the analytical curve. The limits of quantification for the analyzed elements were 0.01 mg/kg for Al, Fe, K, Mg, Na and S and 0.001 mg/kg for the remaining elements. Two readings for each sample were performed, and the concentration values were taken as the mean of the two readings. To check for possible contamination, one blank sample was analyzed for every 11 samples. To calibrate the apparatus, multi-element standards (SCP Science; in 4% nitric acid) were prepared with different concentrations of inorganic elements, taking UNE-EN ISO 11885 as a reference for the determination of elements by ICP atomic emission spectroscopy. In addition, intermediate patterns of all elements were prepared. The equipment calibration was established per batch with a minimum of five points for each lot. Each run began with the calibration standards, continued with samples alternating with intermediate patterns, and finished with a series of intermediate patterns (≤10% variation coefficient).

#### Determination of VOCs

The determination of VOCs was performed using a SPME fiber to extract and a GC (8000 series) with FID to fingerprint the VOCs. Samples of S-MO, N-PO, or the culture medium prior and five days after being in contact with the oils were accurately weighed (2 g), placed in 20-mL glass vials, tightly capped with polytetrafluoroethylene septa, placed in a Combi-pal auto sampler (CTC Analytics AG, Zwingen, Switzerland), and left for 10 min at 40 °C to allow the VOCs in the headspace to equilibrate. The temperature and time were automatically controlled by Varian Star Chromatography Workstation v6.41 (Varian, Walnut Creek, CA, USA). After the equilibrium time, the septum covering each vial was pierced with an SPME needle, and the SPME fiber (1-cm length and 50/30-μm film thickness; Supelco, Bellefonte, PA, USA) was exposed to the headspace for 40 min at 40 °C for VOC extraction. The fiber was endowed with the Stable Flex stationary phase of divinylbenzene/carboxen/polydimethylsiloxane (DVB/CAR/PDMS), and it was previously conditioned according to the supplier’s instructions. The SPME fiber was inserted into the injector port of the GC; the VOCs adsorbed by the fiber were thermally desorbed in the hot GC injection port for 5 min at 260 °C with the purge valve off (splitless mode) and deposited onto a TR-WAX capillary column (60 m × 0.25 mm i.d., 0.25 coating; Teknokroma, Barcelona, Spain) of a Varian 3900 GC with FID. The carrier gas was hydrogen at a flow rate of 1.5 mL/min. The oven temperature was held at 40 °C for 10 min and was then programmed to rise at 3 °C/min to a final temperature of 200 °C. The signal was recorded and processed with the WorkStation (v6.41) software. Each sample was analyzed in duplicate. The content of each VOC was calculated from the FID area and expressed as area units. A solution of 4-methyl-2-pentanol (1.2 mg/kg) was used as the internal standard to standardize the results of all the analyses. Thus, the amount (mg/kg) of each VOC was computed by relating the peak area of that VOC to the area of the standard and considering the sample weight and response factor of each VOC.

The VOCs were identified by mass spectrometry^43^ using the GC conditions described above. For this analysis, we employed a gas chromatograph 7820 coupled to a quadrupole mass spectrometer Series MSD5975 (Agilent Technologies, Santa Clara, CA) operating at 70 eV in a range of 40 to 300 atomic mass units. Signal analysis was performed using ChemStation software E.02.02.1431 (Agilent Technologies, Palo Alto, CA). The VOCs were verified by comparing their retention times and spectra with those of authentic reference compounds.

### Statistics

The results were statistically analyzed using the IBM SPSS 19 Statistics package (SPSS, Chicago, IL, USA). The mean ± standard deviation (SD) values of binary variables (maturation, penetration, monospermy, cleavage, blastocyst formation and efficiencies) were obtained after calculating the percentage in every drop of each group and in each of the four replicates. Continuous variables were expressed as the mean ± SD of three (oxidation status) or four (elemental measurements and VOCs concentrations) replicates. Pairwise comparisons of the means were performed using unpaired Student’s t-tests corrected for the inequality of variances (Levene’s test). Differences among the means of more than two groups were determined by mixed-model analysis of variance (ANOVA) with the random effect of replicate, followed by the Bonferroni post hoc test. The threshold for significance was set at P < 0.05.

## Electronic supplementary material

Supplementary Tables S1 and S2
