# Supplementary material for: The overlaying oil type influences in vitro embryo production: differences in composition and compound transfer into incubation medium between oils
Source: Sci Rep. 2017 Sep 5;7:10505. doi: 10.1038/s41598-017-10989-5 (PMC5585310; doi:10.1038/s41598-017-10989-5)
Supplement: Supplementary file 1 — Supplementary Tables S1 and S2 [file 41598_2017_10989_MOESM1_ESM.pdf]

Supplementary information:

The overlaying oil type influences in vitro embryo production: differences in composition and compound transfer into incubation medium between oils

Cristina A. Martinez[1,2], Alicia Nohalez[1,2], Inmaculada Parrilla[1,2], Miguel Motas[1,2], Jordi Roca[1,2], Inmaculada Romero[3], Diego L. García-González[3], Cristina Cuello[1,2], Heriberto Rodriguez-Martinez[4], Emilio A. Martinez[1,2*] & Maria A. Gil[1,2]

[1]Faculty of Veterinary Medicine, International Excellence Campus for Higher Education and Research "Campus Mare Nostrum", University of Murcia, Murcia, Spain

[2]Institute for Biomedical Research of Murcia (IMIB-Arrixaca), Murcia, Spain

[3]Instituto de la Grasa, (CSIC), Campus University Pablo de Olavide, Sevilla, Spain

[4]Department of Clinical & Experimental Medicine (IKE), Linköping University, Linköping, Sweden

***Corresponding author:** Emilio A. Martinez. E-mail: emilio@um.es; Tel.: +34 868884734, Fax: +34 868887069

This part contains Supplementary Tables S1 and S2

Supplementary Table S1. In vitro fertilization parameters of porcine oocytes cultured in medium overlaid with different lots of Sigma mineral oil (S-MO) and Nidoil paraffin oil (N-PO).

Group	Lot #	Oocytes (N)	Oocytes (%)		Efficiency[&] (%)
			Penetrated[#]	Monospermic[*]	
S-MO	1	245	72.3 ± 11.2	44.3 ± 6.0	31.8 ± 1.8
	2	186	65.0 ± 8.0	55.0 ± 5.0	35.6 ± 3.9
	3	198	73.3 ± 10.4	53.3 ± 6.5	38.7 ± 1.4
N-PO	1	215	65.0 ± 9.6	57.7 ± 7.5	37.4 ± 6.7
	2	205	70.3 ± 11.9	51.8 ± 10.4	36.8 ± 3.3
	3	211	69.0 ± 10.8	52.0 ± 7.1	35.5 ±4.6

[#]Number of oocytes penetrated/total inseminated oocytes. [*]Number of oocytes containing only one male pronucleus/total of oocytes penetrated. [&]Number of monospermic oocytes/total of oocytes inseminated. Data are presented as the mean ± SD (four replicates).

Supplementary Table S2. In vitro fertilization parameters of porcine oocytes cultured in medium overlaid with different lots of Sigma mineral oil (S-MO) and Nidoil paraffin oil (N-PO).

Group	Lot #	Oocytes (N)	Embryo development (%)		Blastocyst efficiency[&] (%)
			Cleavage[#]	Blastocyst formation[*]	
S-MO	1	325	56.8 ± 6.2[a]	49.8 ±5.0[a]	31.8 ± 1.8[a]
	2	356	52.0 ± 5.2[a]	51.7 ± 7.4[a]	35.6 ± 3.9[a]
	3	321	55.3 ± 4.7[a]	49.7 ± 5.9[a]	38.7 ± 1.4[a]
N-PO	1	331	69.5 ± 4.4[b]	69.5 ± 5.8[b]	48.4 ± 6.4[b]
	2	339	65.7 ± 4.2[b]	72.2 ± 7.6[b]	47.6 ± 7.6[b]
	3	319	69.5 ± 4.1[b]	70.5 ± 8.4[b]	49.2 ± 8.2[b]

[#]Number of blastocysts/ total of 2,4-cells embryos. *Number of blastocyst/total of oocytes cultivated. Data are presented as the mean ± SD (four replicates). [a,b] Different letters in the same column indicate differences ($P < 0.05$).
